# Association of Nutritional Status and Possible Sarcopenia Among Formerly Older Homeless Adults in Supportive Housing, Thailand

**DOI:** 10.3390/nu17111776

**Published:** 2025-05-23

**Authors:** Phatcharaphon Whaikid, Noppawan Piaseu

**Affiliations:** 1 Doctor of Philosophy Program in Nursing Science (International Program), Ramathibodi School of Nursing, Faculty of Medicine Ramathibodi Hospital, and Faculty of Nursing, Mahidol University 270 Rama VI Road, Ratchathewi, Bangkok 10400, Thailand; phatcharaporn.wha@hcu.ac.th; 2 Center for Health Promotion and Well-Being, Faculty of Medicine Ramathibodi Hospital, Mahidol University 270 Rama VI Road, Ratchathewi, Bangkok 10400, Thailand

**Keywords:** supportive housing, nutrition, older adults, risk factors, possible sarcopenia

## Abstract

Background/Objectives: Possible sarcopenia and malnutrition are critical public health concerns among older adults, particularly in vulnerable populations such as those with a history of homelessness. However, limited evidence exists on the nutritional status and muscle health of this group in Thailand. Methods: This study aimed to assess the nutritional status of formerly homeless older adults residing in supportive housing in Thailand and examine its association with possible sarcopenia. A cross-sectional study was conducted among 116 participants aged 50 years and older. Sociodemographic information was collected using a structured questionnaire. Nutritional status was then assessed using the Mini Nutritional Assessment short form (MNA-SF) and body mass index (BMI). Possible sarcopenia was determined based on the Asian Working Group for Sarcopenia (AWGS) 2019 criteria, including low calf circumference, reduced handgrip strength, and/or low gait speed. Data were analyzed using descriptive statistics and logistic regression. Results: Of all participants, 66.4% were male, with a mean age of 59.14 years (SD = 7.791). A total of 78.4% were identified as having possible sarcopenia. Univariate analysis revealed significant associations between possible sarcopenia and malnutrition (OR = 6.111, 95% CI = 2.104–17.750), low BMI (OR = 16.784, 95% CI = 3.729–75.535), and waist circumference (OR = 0.129, 95% CI = 0.049–0.342). Multivariate logistic regression indicated that malnutrition (OR = 3.429, 95% CI = 1.093–10.763) and low BMI (OR = 11.732, 95% CI = 2.523–54.567) were significant predictors of possible sarcopenia, collectively explaining 33.8% of the variance. Conclusions: The findings underscore a high prevalence of possible sarcopenia among formerly homeless older adults in supportive housing in Thailand and highlight poor nutritional status, particularly low BMI and malnutrition, as a key contributing factor. These results emphasize the importance of early nutritional screening and interventions to prevent or delay sarcopenia in this vulnerable population.

## 1. Introduction

Sarcopenia is an age-related geriatric syndrome characterized by the progressive loss of skeletal muscle mass, strength, and function [1]. This condition imposes significant burdens on individuals, families, and healthcare systems, as it is associated with increased risks of falls and fractures [2,3], reduced quality of life [4], and higher mortality rates [5]. It is, therefore, considered a major public health concern in aging populations. In 2019, the Asian Working Group for Sarcopenia (AWGS) introduced the concept of “possible sarcopenia” to encourage early identification and prevention [6]. This refers to older adults who exhibit reduced muscle strength and/or physical performance but do not yet meet the diagnostic criteria for sarcopenia. The assessment focuses on these functional indicators, allowing for early detection and timely intervention, such as nutritional support and physical rehabilitation, to prevent further muscle deterioration and functional decline [6]. The prevalence of possible sarcopenia has been reported to range from 46.0% to 68.4% among older adults [7,8].

Older adults experiencing homelessness are known to undergo premature biological aging, often showing age-related decline earlier than the general population. Therefore, individuals aged 50 years and older are commonly classified as older adults in this population context [9]. This early onset of biological aging is closely linked to sarcopenia, with malnutrition being one of the key contributing factors. Older adults with a history of homelessness are especially vulnerable to malnutrition, as a result of prolonged food insecurity and inadequate dietary quality. Even after transitioning to stable housing, many continue to experience nutritional challenges associated with functional limitations, inadequate life skills, and economic hardship [10,11]. Although food assistance is often available, it frequently consists of highly processed and nutrient-poor foods that fail to meet dietary needs and may contribute to chronic health conditions [12].

Malnutrition in this population affects not only general health but also muscle health. Inadequate intake or absorption of essential nutrients, such as proteins, vitamins, and minerals, can lead to significant changes in body composition, particularly loss of muscle mass and body cell mass [13]. Previous research has demonstrated that malnutrition is closely associated with reductions in muscle mass [14], strength, and physical function [15], which are central components of possible sarcopenia. In older adults, nutritional status plays a vital role in maintaining immune function, managing chronic conditions, and preserving physical performance [16].

Similarly, in Thailand, there is growing concern about the increasing number of older adults experiencing homelessness and their elevated risk of malnutrition and related health complications. Although supportive housing provides stability, formerly homeless older adults may remain at risk of poor nutritional status due to accumulated health deficits, unhealthy eating habits, and underlying medical conditions. Given the established association between malnutrition and muscle deterioration, evaluating nutritional status in this population is essential. Early identification is particularly important, as long-term nutritional deprivation, physical inactivity, and comorbidities may accelerate muscle loss. However, data on formerly homeless individuals residing in supportive housing remain limited.

The present study investigates the nutritional status of formerly homeless older adults living in supportive housing in Thailand and examines its association with possible sarcopenia. The findings may inform targeted interventions and policy strategies to improve muscle health, physical function, and overall well-being in this vulnerable population.

## 2. Materials and Methods

### 2.1. Study Design

We conducted this cross sectional study as a part of the main mixed methods study. Ethical approval for the study was obtained from the Ethics Committee of the Faculty of Medicine, Ramathibodi Hospital, Mahidol University, Bangkok, Thailand (MURA2024/64, MURA2025/436). Between December 2024 and February 2025, participants were recruited from the supportive housing facilities where they resided, based on predefined inclusion criteria.

### 2.2. Participants

Participants were eligible for inclusion if they met the following criteria: (1) aged 50 years or older, in accordance with diagnostic criteria commonly used for identifying homelessness in older adults [9]; (2) currently residing, having been there for at least one year, in the Home for the Destitute, Nonthaburi, Thailand; and (3) capable of understanding and communicating in Thai. Participants were excluded if they had a Chula Mental Test (CMT) score < 15 [17], indicating cognitive impairment, and/or an Activities of Daily Living (ADL) score ≤ 8 [18], as significant cognitive decline or loss of autonomy could interfere with accurate assessment of nutritional status and muscle mass.

**Figure 1:**
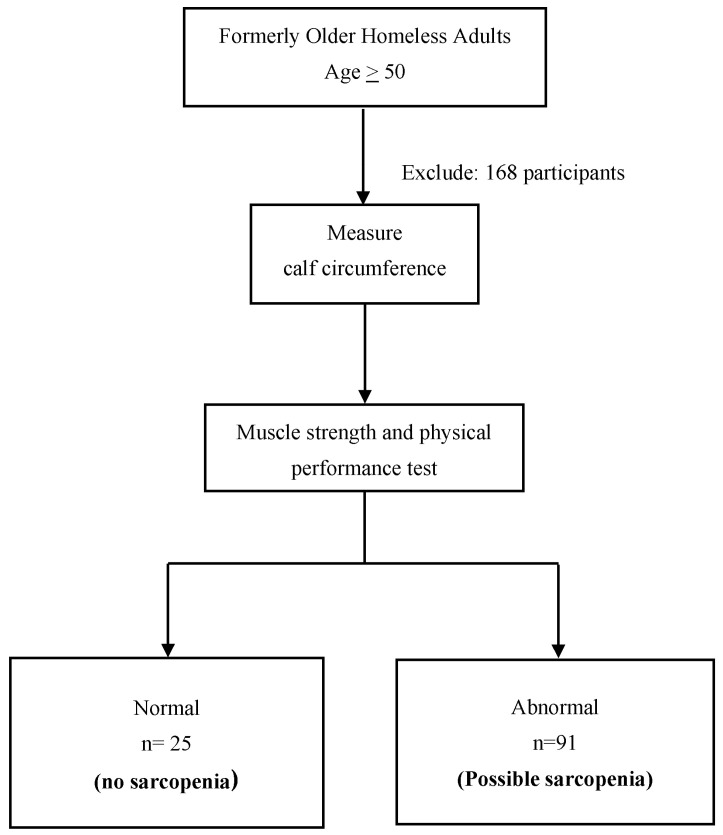
Study design flow diagram.

A total of 284 formerly homeless adults aged 50 years and older were initially screened for eligibility. Of these, 168 individuals were excluded based on predefined criteria, including cognitive impairment, loss of autonomy, or refusal to participate. The remaining 116 participants underwent measurement of calf circumference. Those with low calf circumference proceeded to further evaluation of muscle strength and physical performance using handgrip strength and gait speed assessments, based on the AWGS 2019 criteria. Following these assessments, 91 participants (78.4%) were classified as having possible sarcopenia, while 25 participants (21.6%) had normal muscle strength and physical performance and were categorized as not having sarcopenia (Figure 1).

### 2.3. Data Collection

#### 2.3.1. Data Collection Tools

1. *Screening for possible Sarcopenia*

The assessment of possible sarcopenia was conducted based on the criteria of the Asian Working Group for Sarcopenia (AWGS) 2019. The evaluation began with the measurement of calf circumference, followed by assessments of muscle strength and physical performance as the primary diagnostic indicators [6].

- Assessment of Calf circumference

A calf was measured using a non-stretchable measuring tape while the participant was sitting in a relaxed position. A circumference of less than 34 cm for men and less than 33 cm for women was considered indicative of risk for sarcopenia [6].

- Assessment of Muscle Strength

Muscle strength was assessed using a digital hand dynamometer to measure handgrip strength. Cutoff values of less than 28 kg for men and less than 18 kg for women were applied as indicators of low muscle strength, in accordance with the AWGS 2019 recommendations [6].

- Assessment of a 6 m gait speed test was used to assess physical performance

Gait speed measurement is a simple and practical method for assessing physical performance. In this study, gait speed was assessed over a 6 m distance using a stopwatch, with participants walking at their usual pace. According to the AWGS 2019 criteria, a cutoff value of <1.0 m/s was used to indicate low gait speed. The time taken to complete the distance was recorded to the nearest 0.01 s [6].

2. *Nutritional Status*

The nutritional status in this study was assessed using two indicators: the Mini Nutritional Assessment short form (MNA-SF) and body mass index (BMI). The MNA-SF is a validated seven-item screening tool recommended by the European Society for Clinical Nutrition and Metabolism (ESPEN) [19], with a total score ranging from 0 to 14. Scores of 12–14 indicate normal nutritional status, whereas scores below 12 suggest a risk of malnutrition or the presence of malnutrition. BMI was calculated by dividing body weight (kg) by the square of height (m^2^). A cutoff point of 20 was applied, as it has been shown to better differentiate underweight from normal weight in older adults [20].

3. *Sociodemographic and health-related information*

A general information questionnaire was used to collect participants’ sociodemographic and health-related characteristics. Variables included age, gender, BMI, waist circumference, and comorbidity status.

#### 2.3.2. Data Collection Process

The researcher trained 12 research assistants (RAs) to support data collection. The physical flow of data collection involved participants moving sequentially through five assessment stations.

Station 1 involved cognitive screening using the Chula Mental Test and an Activities of Daily Living (ADL) assessment. A total of 168 participants did not meet the inclusion criteria. The remaining eligible participants proceeded to Station 2 (~5 min), where they were interviewed regarding demographic characteristics. At Station 3 (~5 min), participants were asked to stand on a digital scale to measure body weight and then had their height measured using a stadiometer. While seated, their calf circumference was measured, followed by waist circumference. Station 4 (~2 min) involved participants sitting on a chair and squeezing a digital dynamometer twice with both their left and right hands at maximum strength. At Station 5 (~2 min), participants walked six meters to assess gait speed.

#### 2.3.3. Statistical Analysis

All statistical analyses were performed using SPSS version 21.0 (IBM Corp., Armonk, NY, USA). Descriptive statistics were used to summarize participants’ characteristics. Categorical variables were presented as frequencies and percentages, while continuous variables were expressed as means and standard deviations (SDs). Group differences between participants with and without possible sarcopenia were examined using the Chi-square test for categorical variables and independent *t*-tests for continuous variables.

Univariate logistic regression was performed to examine the crude associations between individual variables and possible sarcopenia. Variables with *p*-values < 0.05 in the univariate analysis were included in the multivariate logistic regression model to identify independent predictors. Only nutritional variables (MNA-SF and BMI) met this criterion and were thus retained in the final model. Adjusted odds ratios (AORs), 95% confidence intervals (CIs), and *p*-values were reported. A *p*-value < 0.05 was considered statistically significant.

## 3. Results

The characteristics of the study population show that the mean age of participants in the possible sarcopenia group was significantly higher than that of the non-sarcopenia group (60.34 ± 7.90 vs. 54.76 ± 5.59 years, *p* = 0.001). Although a higher proportion of participants aged ≥ 60 years was observed in the possible sarcopenia group (44.0%) compared to the non-sarcopenia group (24.0%), the difference was not statistically significant (*p* = 0.071). Regarding gender, males represented the majority in both groups, and no significant difference was found between groups (*p* = 0.446). A statistically significant difference in BMI was observed between groups. The possible sarcopenia group had a lower mean BMI (19.73 ± 2.69 kg/m^2^) compared to the non-sarcopenia group (26.44 ± 4.84 kg/m^2^), (*p* < 0.001). Most individuals with BMI < 20 kg/m^2^ (96.4%) and ≥20 kg/m^2^ (61.7%) were in the possible sarcopenia group (*p* < 0.001). For nutritional status assessed using the MNA-SF, participants with scores < 12 (indicating malnutrition or risk of malnutrition) were predominantly in the possible sarcopenia group (91.7%), while only 8.3% were in the non-sarcopenia group (*p* < 0.001). Comorbidity was common in both groups, with no significant difference observed (*p* = 0.407). The mean waist circumference was significantly lower in the possible sarcopenia group compared to the non-sarcopenia group (76.57 ± 8.67 cm vs. 91.76 ± 12.33 cm, *p* < 0.001). A significantly higher proportion of participants with normal waist circumference was observed in the possible sarcopenia group (89.2%) compared to the non-sarcopenia group (10.8%) (*p* < 0.001), while the proportions of high waist circumference were similar between the groups. Regarding frailty status, no statistically significant difference was found between groups (*p* = 0.608). Among those categorized as frail, 66.7% were in the possible sarcopenia group and 33.3% were in the non-sarcopenia group. Similarly, among participants with normal frailty scores, 79.1% were in the possible sarcopenia group and 20.9% in the non-sarcopenia group (Table 1).

Participants in the possible sarcopenia group had a significantly lower mean calf circumference than those in the non-sarcopenia group (30.32 ± 2.00 cm vs. 35.88 ± 2.74 cm, *p* < 0.001). Notably, 97.8% of individuals in the possible sarcopenia group were classified as having low calf circumference, whereas all participants with normal calf circumference belonged to the non-sarcopenia group (*p* < 0.001). Regarding muscle strength, the mean handgrip strength was significantly lower in the possible sarcopenia group (19.14 ± 7.48 kg) than in the non-sarcopenia group (22.30 ± 5.70 kg), with a *p*-value of 0.027. Additionally, 83.3% of participants with low handgrip strength were in the possible sarcopenia group, while only 16.7% were in the non-sarcopenia group (*p* = 0.017). Regarding physical performance, mean gait speed did not differ significantly between groups (0.89 ± 0.29 m/s in the possible sarcopenia group vs. 0.92 ± 0.31 m/s in the non-sarcopenia group, *p* = 0.485). Similarly, the proportion of participants with low gait speed was not significantly different between groups (*p* = 0.901) (Table 2).

Age was significantly associated with higher odds of possible sarcopenia. Specifically, each additional year of age increased the likelihood of possible sarcopenia by 15.4% (OR = 1.154, 95% CI: 1.050–1.268, *p* = 0.003). Waist circumference was another significant factor. Participants with normal waist circumference had significantly lower odds of possible sarcopenia than those with high waist circumference (OR = 0.129, 95% CI: 0.049–0.342, *p* < 0.001). As assessed by the MNA and BMI, nutritional status was also strongly associated with possible sarcopenia. Participants with low BMI had significantly greater odds of possible sarcopenia (OR = 16.784, 95% CI: 3.729–75.535, *p* = 0.001). Moreover, those at risk of malnutrition or classified as malnourished had over sixfold increased odds compared to individuals with normal nutritional status (OR = 6.111, 95% CI: 2.104–17.750, *p* = 0.001). In the multivariate analysis, age remained a significant predictor (OR = 1.152, 95% CI: 1.023–1.299, *p* = 0.020). Similarly, low BMI (OR = 5.315, 95% CI: 1.000–28.266, *p* = 0.050) and being at risk of malnutrition or malnourished according to the MNA-SF (OR = 4.757, 95% CI: 1.170–19.346, *p* = 0.029) were independently associated with increased odds of possible sarcopenia. Normal waist circumference was independently associated with significantly lower odds (OR = 0.181, 95% CI: 0.044–0.741, *p* = 0.017). In contrast, gender, frailty status, and comorbidity were not significantly associated with possible sarcopenia. (Table 3).

Multivariate logistic regression analysis indicated that decreased MNA and low BMI together explained 33.38% of the model’s prediction of possible sarcopenia (Table 4).

## 4. Discussion

This cross-sectional study was the first to assess possible sarcopenia and its association with nutritional status among formerly homeless adults aged 50 years and older, based on the diagnostic criteria proposed by the Asian Working Group for Sarcopenia (AWGS) 2019 [6].

### 4.1. Prevalence of Possible Sarcopenia and Nutrition Status

This study revealed a high prevalence of possible sarcopenia (78.4%) among formerly homeless older adults residing in supportive housing in Thailand. This rate is considerably higher than those reported in community-dwelling older populations in previous studies [7,8], suggesting that formerly homeless individuals may face elevated risks due to long-term nutritional deficits, and accumulated health burdens. Our findings reveal that many formerly homeless individuals residing in supportive housing face considerable challenges related to malnutrition, with 51.72% of participants classified as being at risk of malnutrition or malnourished. This proportion is notably higher than that reported among stably housed older adults [21,22]. Notably, 91.7% of those identified as malnourished were also classified as having possible sarcopenia, underscoring the strong link between poor nutritional status and muscle decline in this vulnerable population.

Furthermore, our study identified age, waist circumference, and nutritional status (including low BMI and malnutrition) as contributing factors to possible sarcopenia. Possible sarcopenia in this study was defined by low calf circumference, reduced muscle strength, or/and impaired physical performance [6]. The association between age and possible sarcopenia aligns with previous research indicating that advancing age is linked to declines in muscle strength [23,24] and physical performance [25,26]. Calf circumference, a simple yet valuable anthropometric indicator, is widely used to estimate muscle mass in older adults. Multiple studies have demonstrated its reliability in detecting age-related muscle loss [27]. Notably, a longitudinal study reported a reduction in calf circumference ranging from 1.1 to 3.4 cm over a 15-year period, with more pronounced decreases observed among the oldest age groups compared to their younger counterparts [28,29]. These findings are consistent with our study, which found that increasing age was significantly associated with a greater likelihood of possible sarcopenia.

Waist circumference was significantly associated with possible sarcopenia. Participants with a normal waist circumference had 87% lower odds of having possible sarcopenia compared to those with high waist circumference. This finding is consistent with previous studies indicating that elevated waist circumference reflects a higher proportion of body fat and greater abdominal obesity [30]. Consistently, other studies have found that increased waist circumference is associated with reduced muscle mass [31] and muscle strength [32].

### 4.2. Association Between Nutritional Status and Possible Sarcopenia

Our findings indicate that possible sarcopenia is associated with poor nutritional status, as reflected by suboptimal MNA scores and low BMI. This result aligns with previous studies suggesting that a lower BMI may serve as a marker of sarcopenia and may be linked to metabolic alterations, such as reduced triglyceride levels [33]. Additionally, nutritional status was significantly associated with possible sarcopenia, reinforcing prior evidence in this area [34]. Notably, multivariate logistic regression analysis revealed that nutritional status and BMI together explained 33.8% of the variance in possible sarcopenia, underscoring the strong predictive value of these factors. These findings are consistent with earlier research demonstrating associations with reduced muscle strength [35] and impaired physical performance [36] and further support the notion that nutritional status may play a critical role in the onset and progression of sarcopenia [37]. Importantly, nutritional status was found to be a significant predictor of possible sarcopenia. Participants with low MNA scores—indicating a risk of malnutrition or existing malnutrition—had more than six times greater odds of having possible sarcopenia compared to those with normal nutritional status. This finding aligns with prior research demonstrating that inadequate intake of protein and essential nutrients contributes to muscle loss, reduced strength, and physical decline in older adults [14,15]. Maintaining muscle health is essential for preserving functional ability and independence in older adults. The previous literature has demonstrated that specific foods, nutrients, and dietary patterns may help prevent or mitigate age-related declines in muscle strength and physical function [38]. This is consistent with findings showing that older adults with higher protein intake tend to exhibit greater stimulation of muscle protein synthesis [39,40]. These physiological benefits are closely tied to body composition. Inadequate nutrient intake and poor dietary quality can accelerate the loss of muscle mass, particularly skeletal muscle, while increasing fat mass [41]. Such imbalances not only impair physical function but also increase the risk of sarcopenia, especially among nutritionally vulnerable populations. Furthermore, sarcopenia is not only a marker of frailty but is also strongly associated with adverse outcomes such as falls. Previous evidence identifies sarcopenia as a modifiable risk factor for fall-related injuries, including fractures, disability, and loss of independence. Declines in muscle mass and strength heighten vulnerability to slips and trips [42,43,44]. Sarcopenia is also associated with cardiovascular diseases, particularly heart failure and arrhythmias, where its prevalence is significantly higher than in the general population [45,46].

Given its wide-ranging impact on health outcomes, sarcopenia represents an emerging public health concern. Early detection and nutritional interventions could play a key role in reducing healthcare burdens and informing preventive health policies. Therefore, promoting optimal dietary protein intake may be an effective intervention to support muscle maintenance and functional independence in aging populations.

One of the key strengths of this study is its focus on a vulnerable and understudied population, formerly homeless older adults living in supportive housing. Using standardized and validated tools such as MNA, handgrip dynamometry, gait speed testing, and AWGS 2019 criteria ensures a reliable nutritional status and muscle health assessment. Moreover, the study provides novel evidence from the Thai context, where data on sarcopenia in this specific population are lacking.

However, some limitations should be considered. First, the cross-sectional design precludes causal inference between nutritional status and possible sarcopenia. Second, the study was conducted in a single facility in Thailand, which may limit the generalizability of the findings to other settings or countries. Third, the exclusion of participants with cognitive impairment may have led to an underestimation of sarcopenia prevalence, as this group may be at even higher risk. Finally, this study did not assess other factors that may influence sarcopenia, such as physical activity level or inflammatory biomarkers.

## 5. Conclusions

This study found a high prevalence of possible sarcopenia among formerly homeless older adults residing in supportive housing in Thailand. Poor nutritional status (low MNA and low BMI) was significantly associated with possible sarcopenia. Together, these factors explained 33.8% of the variation in sarcopenia risk. The findings highlight the importance of routine screening for nutritional status and muscle health in vulnerable populations. Early identification and the development of integrated care strategies focusing on nutritional support and physical activity are essential to prevent muscle deterioration and promote long-term health and quality of life among formerly homeless older adults. Possible strategies to prevent sarcopenia among formerly older homeless adults residing in supportive housing must consider the unique constraints of these institutional settings. Given the limited resources, staff, and individualized services, simple and cost-effective approaches may be most practical. These could include the provision of protein-rich meals tailored to older adults’ nutritional needs, basic resistance or functional exercises incorporated into daily routines, and periodic screening for sarcopenia. As these facilities are governed by the Department of Social Development and Welfare, integrating sarcopenia prevention into existing care routines, such as collaboration with staff, would enhance feasibility and sustainability.

## Figures and Tables

**Table 1:** Participant characteristics and nutritional status.

Sociodemographic Characteristics		Total (n)	Non-Sarcopenia	Possible Sarcopenia	*p*-Value
**All participants**, n (%)		116	25 (21.6%)	91 (78.4%)	
**Age (years)**, mean ± SD		116	54.76 ± 5.59	60.34 ± 7.90	0.001
**Age**, n (%)					
	50–59	70	19 (76.0%)	51 (56.0%)	
	≥60	46	6 (24.0%)	40 (44.0%)	0.071
**Gender**, n (%)					
	Female	39	10 (25.6%)	29 (74.4%)	0.446
	Male	77	15 (19.5%)	62 (80.5%)	
**Body mass index** (kg/m^2^), mean ± SD		116	26.44 ± 4.84	19.73 ± 2.69	<0.001
**Body mass index**, n (%)					
	<20 kg/m^2^	56	2 (3.6%)	54 (96.4%)	<0.001
	≥20 kg/m^2^	60	23 (38.3%)	37 (61.7%)	
**MNA-SF, n (%)**					
	<12	60	5 (8.3%)	55 (91.7%)	<0.001
	≥12	56	20 (35.7%)	36 (64.3%)	
**Comorbidity**, n (%)					
	No	15	2 (13.3%)	13 (86.7%)	0.407
	Yes	101	23 (22.8%)	78 (77.2%)	
**Waist circumference** (cm), mean ± SD		116	91.76 ± 12.33	76.57 ± 8.67	<0.001
**Waist circumference**, n (%)					
	High	33	16 (48.5%)	17 (51.5%)	
	Normal	83	9 (10.8%)	74 (89.2%)	<0.001
**Frailty**, n (%)					
	Low	6	2 (33.3%)	4 (66.7%)	
	Normal	110	23 (20.9%)	87 (79.1%)	0.608

**Table 2:** Calf circumference, muscle strength, and physical performance in the study participants.

Sociodemographic Characteristics		Total (n)	Non-Sarcopenia	Possible Sarcopenia	*p*-Value
**Calf circumference** (cm),		116	35.88 ± 2.74	30.32 ± 2.00	<0.001
mean ± SD					
**Calf circumference**, n (%)					
	Low	93	2 (2.2%)	91 (97.8%)	
	Normal	23	23 (100%)	-	<0.001
**Handgrip strength** (kg),		116	22.30 ± 5.70	19.14 ± 7.48	0.027
mean ± SD					
**Handgrip strength**, n (%)					
	Low	90	15 (16.7%)	75 (83.3%)	
	Normal	26	10 (38.5%)	16 (61.5%)	0.017
**Gait speed** (m/s), mean ± SD		116	0.92 ± 0.31	0.89 ± 0.29	0.485
**Gait speed**, n (%)					
	Low	43	9 (20.9%)	34 (79.1%)	
	Normal	73	16 (21.9%)	57 (78.1%)	0.901

**Table 3:** Nutritional status and factors associated with possible sarcopenia.

Variables		Crude OR	95% CI	*p*-Value	Adjusted OR	95% CI	*p*-Value
**Age**		1.154	1.050–1.268	0.003	1.152	1.023–1.299	0.020
**Gender**							
	Female *						
	Male	0.702	0.281–1.749	0.447			
**Body mass index**							
	<20 kg/m^2^	16.784	3.729–75.535	<0.001	5.315	1.000–28.266	0.050
	≥20 kg/m^2^ *						
**MNA-SF**							
	Normal nutritional status *						
	At risk of malnutrition or malnourished	6.111	2.104–17.750	0.001	4.757	1.170–19.346	0.029
**Waist circumference**							
	High						
	Normal *	0.129	0.049–0.342	<0.001	0.181	0.044–0.741	0.017
**Frail**							
	Low *	0.529	0.091–3.069	0.478			
	Normal						
**Comorbidity**							
	No *						
	Yes	0.522	0.110–2.482	0.414			

* Reference group; OR = odds ratio; CI = confidence interval.

**Table 4:** Association between sarcopenia and nutritional status.

Variables		Nagelkerke R-Square	Adjusted OR	95% CI	*p*-Value
**Possible Sarcopenia**					
	MNA < 12	0.338	3.429	1.093–10.763	0.035
	Low BMI		11.732	2.523–54.567	0.002

OR = odds ratio; CI = confidence interval

## Data Availability

The data that support the findings of this study are available from the corresponding author upon reasonable request. The data are not publicly available due to privacy issues.
